# Supplementary material for: Reversible photoregulation of cell-cell adhesions with opto-E-cadherin
Source: Nat Commun. 2023 Oct 9;14:6292. doi: 10.1038/s41467-023-41932-0 (PMC10562482; doi:10.1038/s41467-023-41932-0)
Supplement: Supplementary file 1 — Supplementary Information [file 41467_2023_41932_MOESM1_ESM.pdf]

Supplementary Information

Reversible Photoregulation of Cell-Cell Adhesions with Opto-E-cadherin

Brice Nzigou Mombo,[1] Brent M. Bijonowski,[1] Christopher A. Raab,[1] Stephan Niland,[1] Katrin Brockhaus,[1] Marc Müller,[1] Johannes A. Eble,[1] Seraphine V. Wegner[1,]*

[1] Institute of Physiological Chemistry and Pathobiochemistry, University of Münster, Waldeyerstraße 15, 48149 Münster, Germany.

*E-Mail: wegnerse@uni.muenster.de

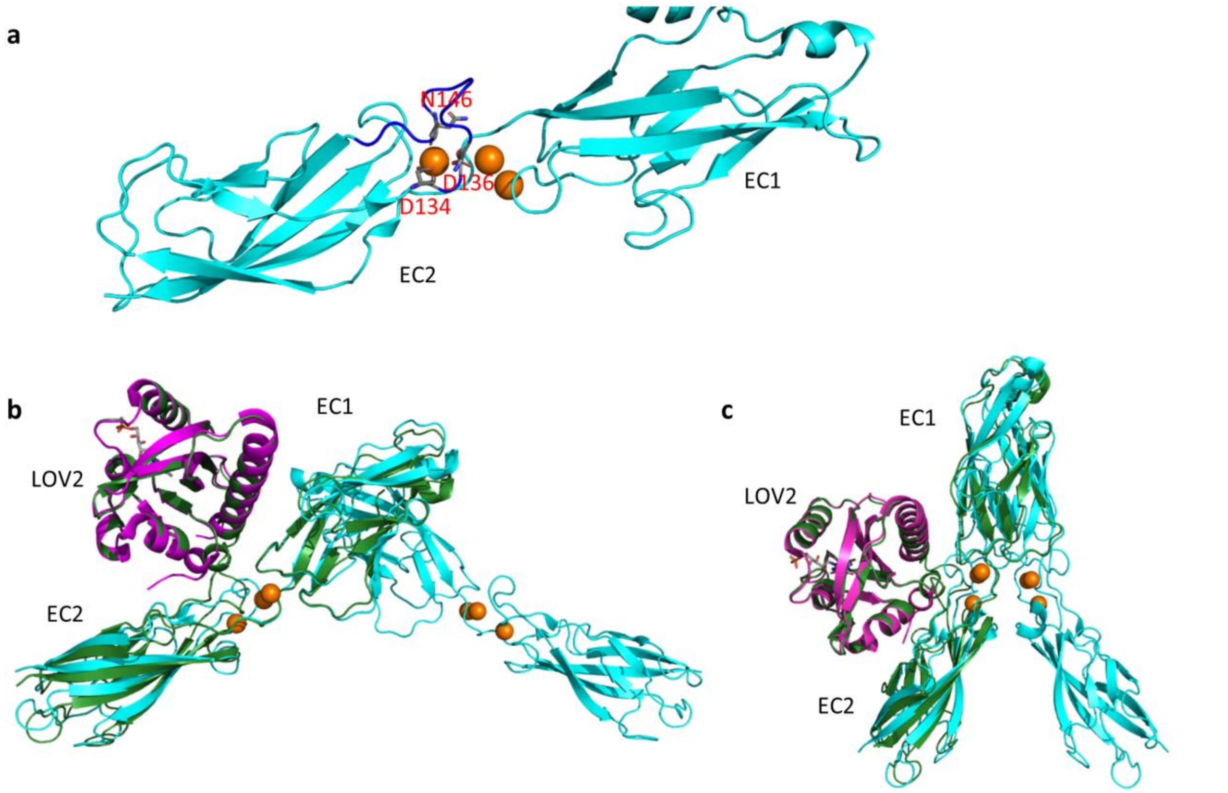

Supplementary Fig. 1 | Molecular design of Opto-E-cad. a, Ca^{2+} (shown in orange) binding site in E-cadherin (PDB: 2O72). The LOV2 domain was inserted into the loop (shown in blue) after T133 as it includes the ligands for two of the Ca^{2+} ions in the side chains of D134 and D136 as well as the backbone of N146. Structure of opto-E-cad (shown in green) as modelled by AlphaFold overlaid with **b,** the trans-dimer (PDB: 2O72) and **c,** the cis-dimer (PDB: 4ZT1) of human E-cadherin (shown in cyan) as well as the LOV2 domain in the dark (PBD: 2V1A) (shown in pink). The LOV2 domain was inserted in loop (D134-I146) of EC2 such that it would not interfere with the E-cadherin cis- or trans-dimerization.

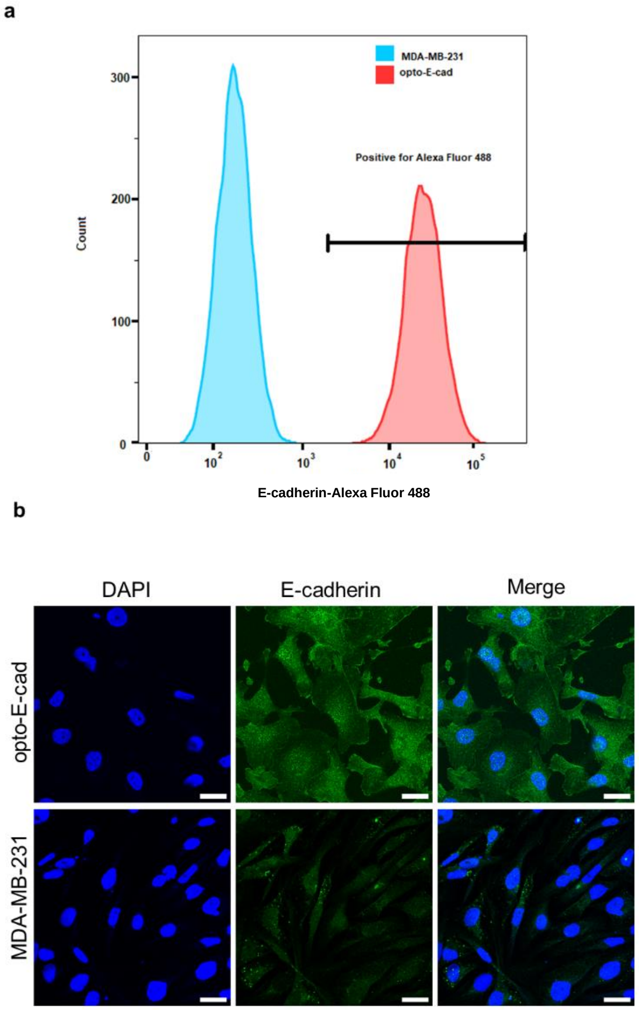

Supplementary Fig. 2 | Opto-E-cad expression at the plasma membrane. a, FACS analysis of opto-E-cad-MDA and MDA-MB-231 cells stained with E-cadherin antibody. **b,** Confocal fluorescence microscopy images of the opto-E-cad-MDA and MDA-MB-231 cells stained with DAPI (shown in blue) and E-cadherin antibody (shown in green). Scale bars are 25 µm, n=1.

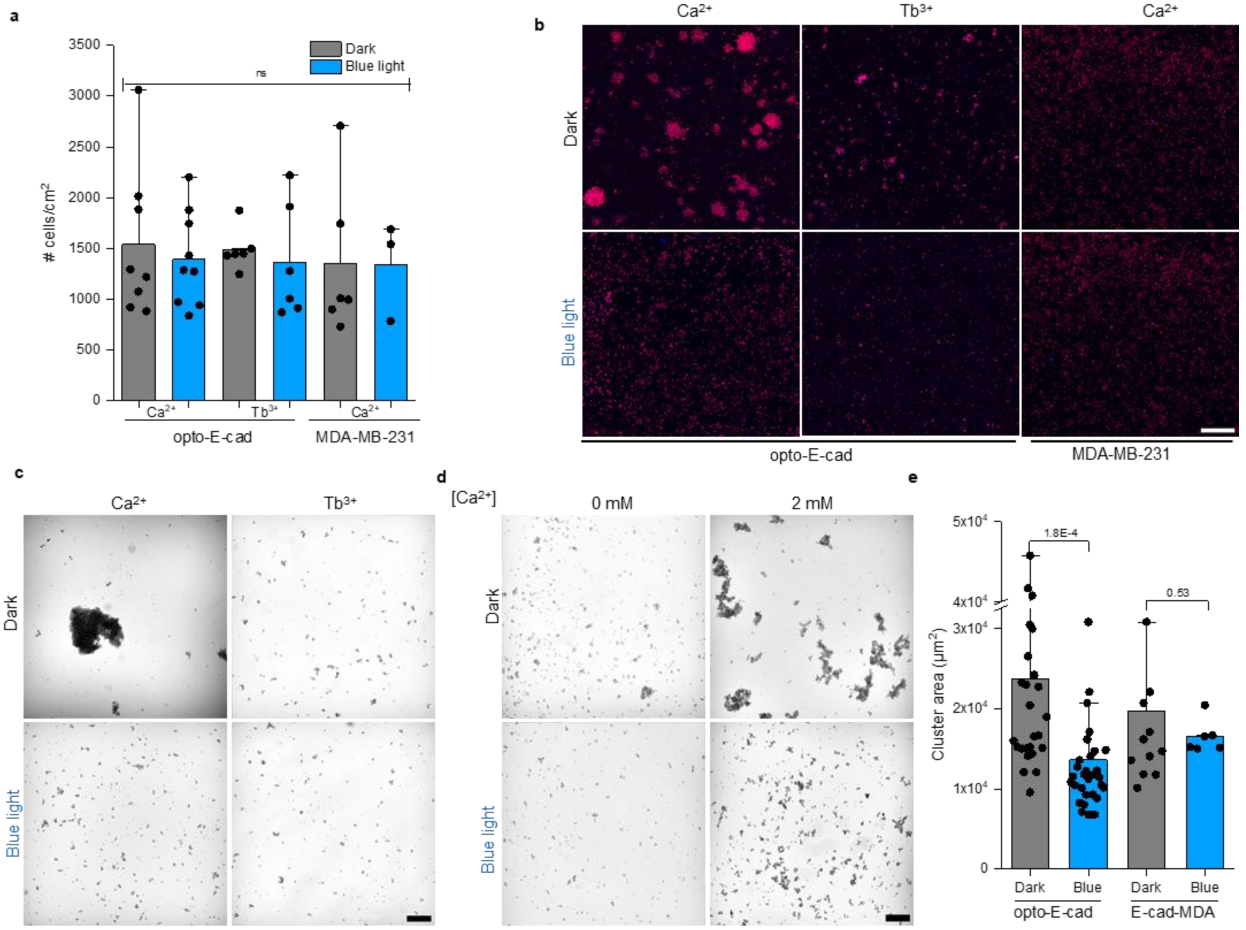

Supplementary Fig. 3 | Light and calcium response of opto-E-cad-MDA cells. a, Cell seeding density in 2D cultures in Fig. 1c as determined by nuclei staining (n≥3). All comparisons are performed using Fisher's One Way ANOVA test and $p<0.05$ was treated as the significance threshold. **b,** Low magnification fluorescence microscopy images of opto-E-cad-MDA and MDA-MB-231 cells on glass surfaces after 4 h in the dark or under blue light (n=3). Actin shown in red; nuclei shown in blue. Scale bar is 1 mm. **c,** Bright field microscopy images of opto-E-cad-MDA cells in suspension culture in the presence of Ca^{2+} or Tb^{3+}(n=3). Scale bar is 100 µm. **d,** Bright field microscopy images of opto-E-cad-MDA cells in suspension culture in the presence or absence of Ca^{2+} (2 mM) (n=3). Scale bar is 100 µm. **e,** Quantification of average cluster area for opto-E-cad-MDA and E-cad-MDA cells in the dark and under blue light (two-tailed t-test, from left to right n=29, 32, 12, 6). Data are presented as mean values ± SD. Source data are provided as a Source Data file.

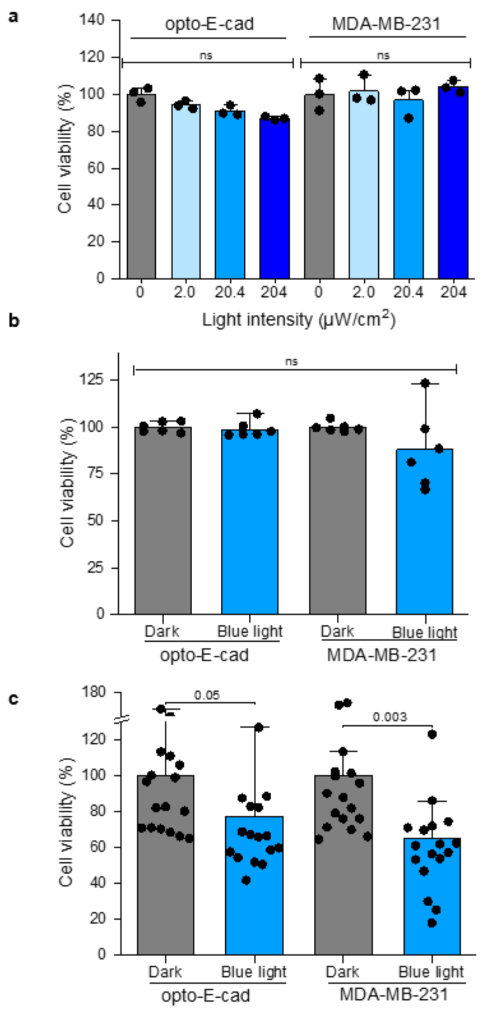

Supplementary Fig. 4 | Cell viability under different light conditions. **a,** Cell viability for opto-E-cad-MDA and wild-type MDA-MB-231 cells incubated in 2D cell cultures in the dark and under different blue light (463 nm) intensities (LED light module V10 with TS-110 Controller, CLF Plant Climatics GmbH) for 24 h (n=3). These conditions were used for cell signalling studies (immunofluorescence and western blot) and the invasion assay. **b,** Cell viability for opto-E-cad-MDA and wild-type MDA-MB-231 cells in the Transwell invasion assay. Cells were incubated under blue light (463 nm, 20.4 µW/cm^2) overnight (n=6). **c,** Cell viability for opto-E-cad-MDA and wild-type MDA-MB-231 cells during the migration assay under blue light (272 µW/cm^2) or in the dark (n=18). Data are presented as individual values ± SD. All comparisons are performed using Fisher's One Way ANOVA and $p<0.05$ was treated as the significance threshold. Afterwards, cell viability was measured by the MTT assay for differences between (a), (b) and (c). Source data are provided as a Source Data file.

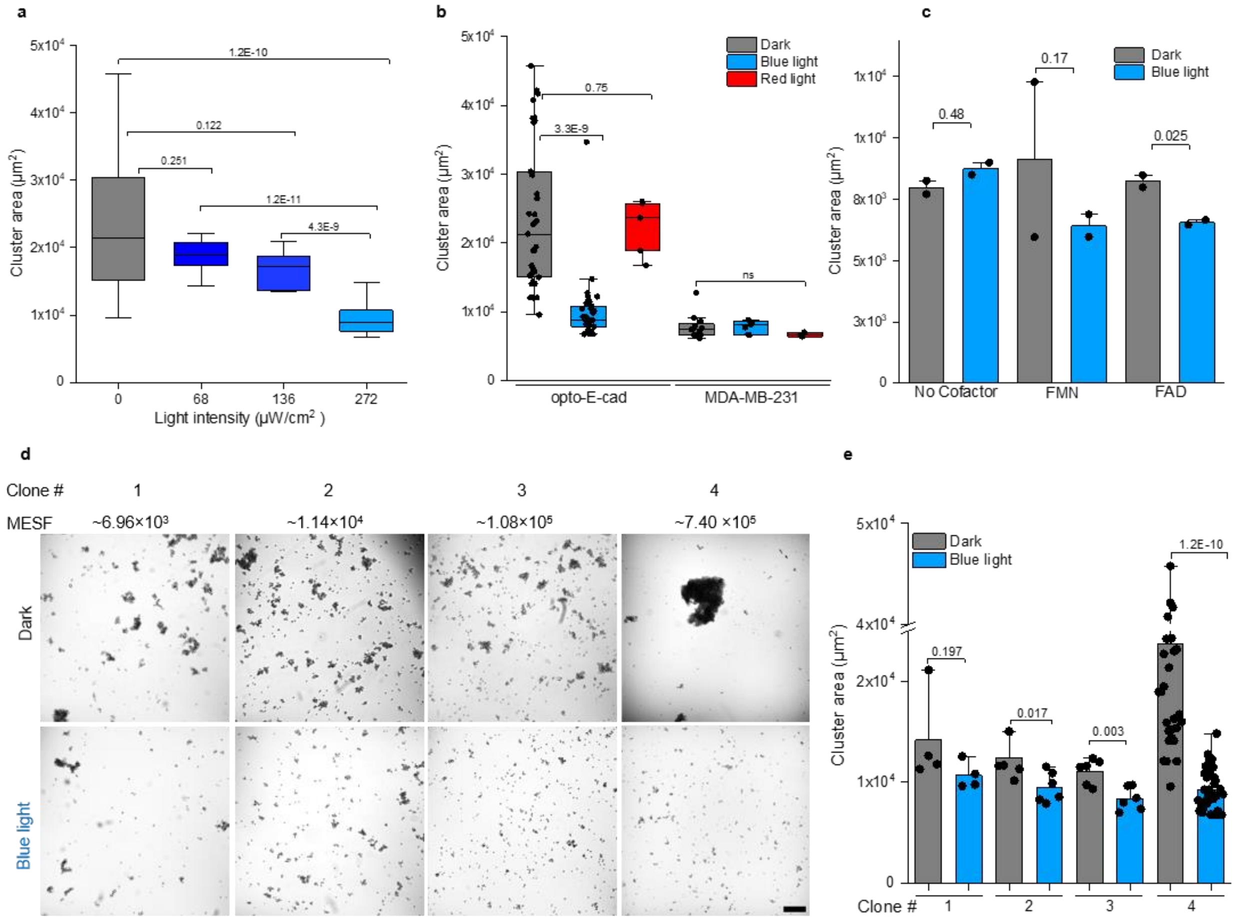

Supplementary Fig. 5 | Performance of opto-E-cad depending on light intensity, wavelength and expression level of opto-E-cad. Cell aggregation assay with opto-E-cad-MDA cells with **a,** different light intensities (n=6), **b,** different light wavelengths (n=6) and **c,** in the absence or presence of cofactor (n=2). All comparisons are performed using two-tailed t-test and $p<0.05$ was treated as the significance threshold. **d,** Opto-E-cad expression levels on different opto-E-cad-MDA monoclonal cell lines observed by using the Molecule of Equivalent Soluble Fluorochrome (MESF) kit and bright field images of different opto-E-cad-MDA clones in the dark and under blue light. Scale bar is 100 µm. Clone #4 was used in all other experiments with opto-E-cad-MDA cells. **e,** Quantification of average cluster area for different opto-E-cad-MDA clones (two-tailed t-test, n≥4). Data are presented as mean values ± SD. Source data are provided as a Source Data file.

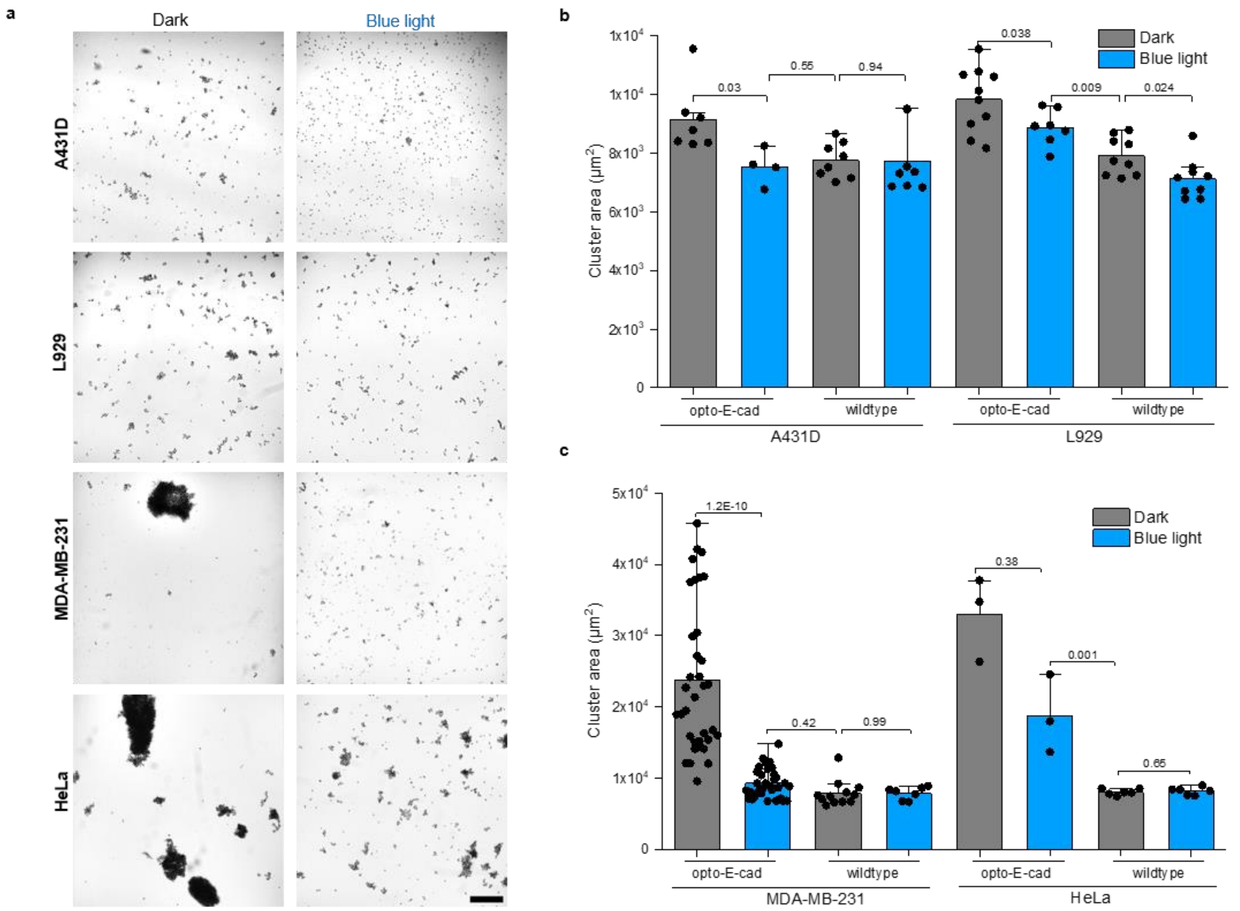

Supplementary Fig. 6 | Opto-E-cad in different cell lines. a, Bright field images of stable opto-E-cad cell lines established from A431D, L929, MDA-MB-231 and HeLa cells in the dark and under blue light. Scale bar is 100 µm. Quantification of average cluster area for **b,** A431D and L929 cells with and without opto-E-cad (n≥4) and **c,** MDA-MB-231 and HeLa cells with and without opto-E-cad (n≥3). Data are presented as mean values ± SD. All comparisons are performed using two-tailed t-test and $p<0.05$ was treated as the significance threshold. Source data are provided as a Source Data file.

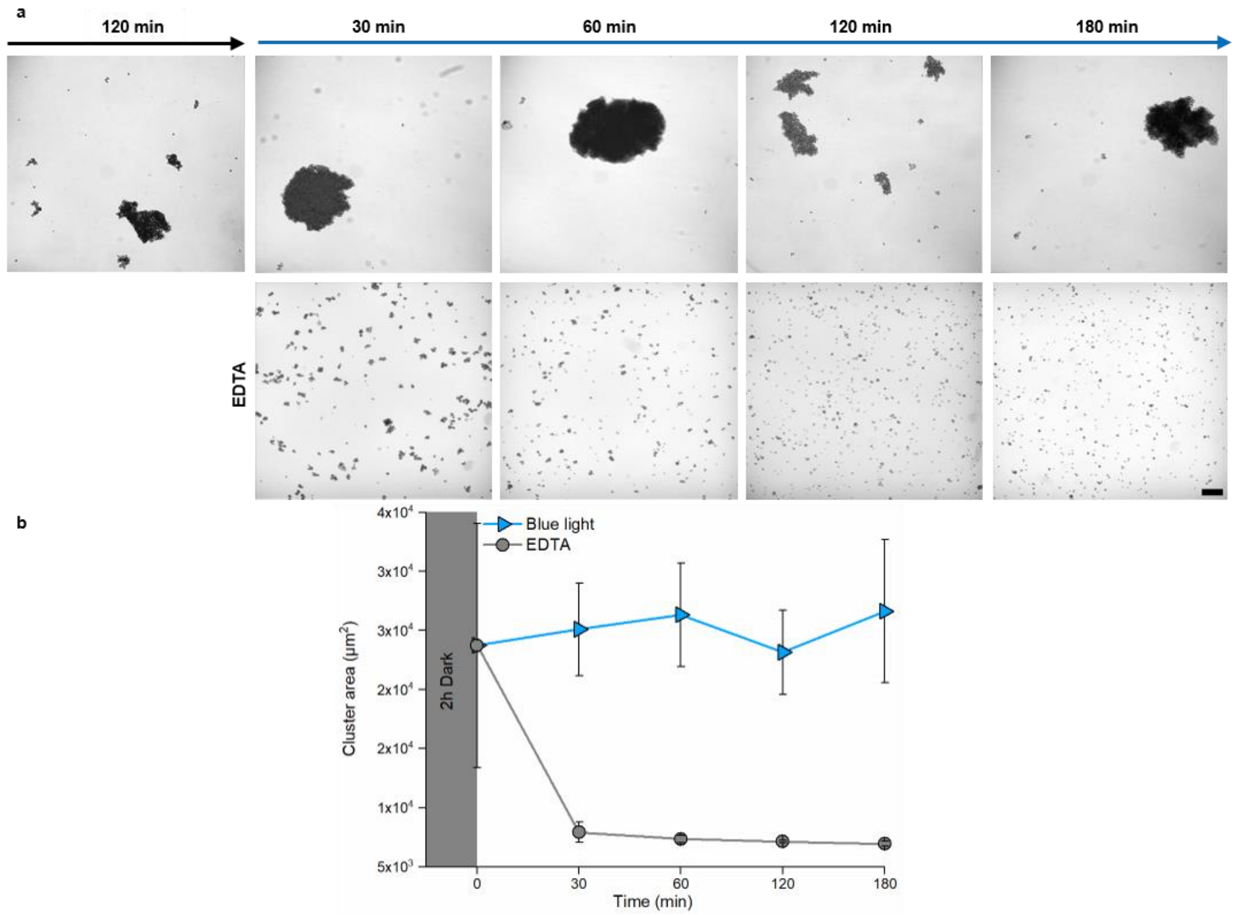

Supplementary Fig. 7 | Reversibility of the cell-cell adhesions after 120 min preincubation in the dark and subsequent blue light illumination. **a,** Bright field images of opto-E-cad-MDA cells in suspension culture kept for 120 min in the dark and subsequently for 180 min under blue light or in the presence of EDTA (3 mM). Scale bar is 200 µm. **b,** Average cluster size over time for samples in a (n≥3). Data are presented as mean values ± SD. Source data are provided as a Source Data file.

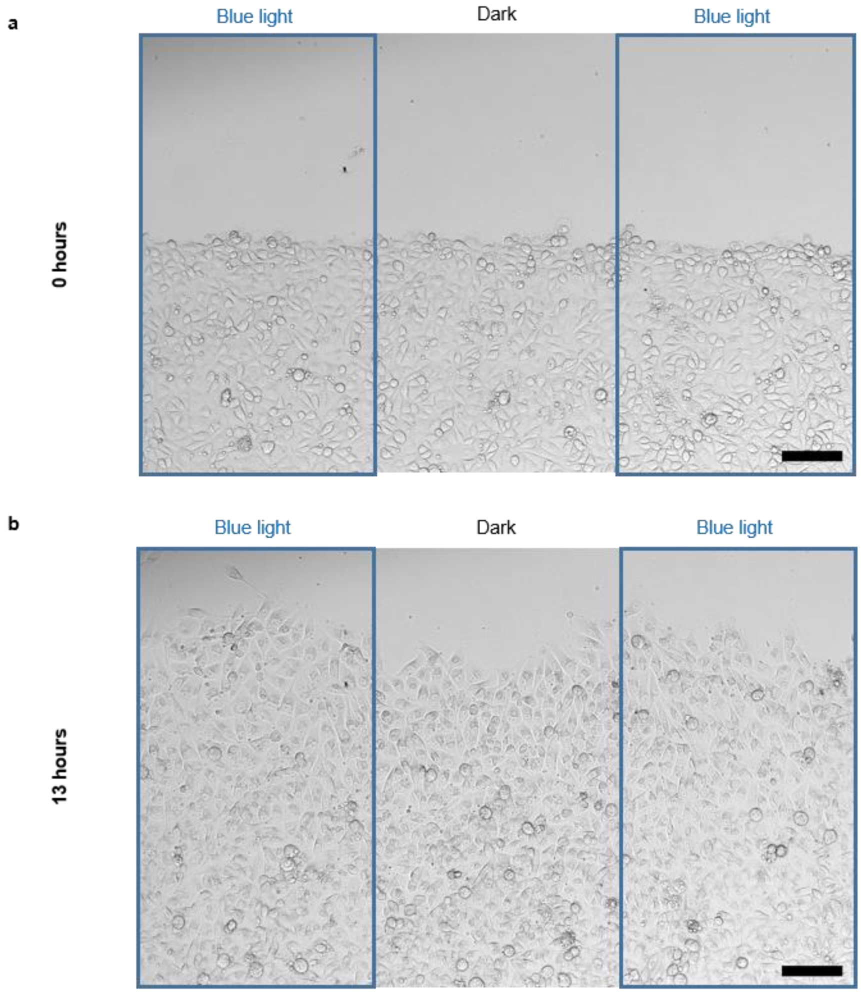

Supplementary Fig. 8 | Spatiotemporal control in the wound healing assay with opto-E-cad-MDA cells. Internal reflection images of the wound healing assay at **a,** 0 h and **b,** 13 h, where parts of the wound were illuminated (shown as a blue box, n=1). Scale bar is 100 µm.

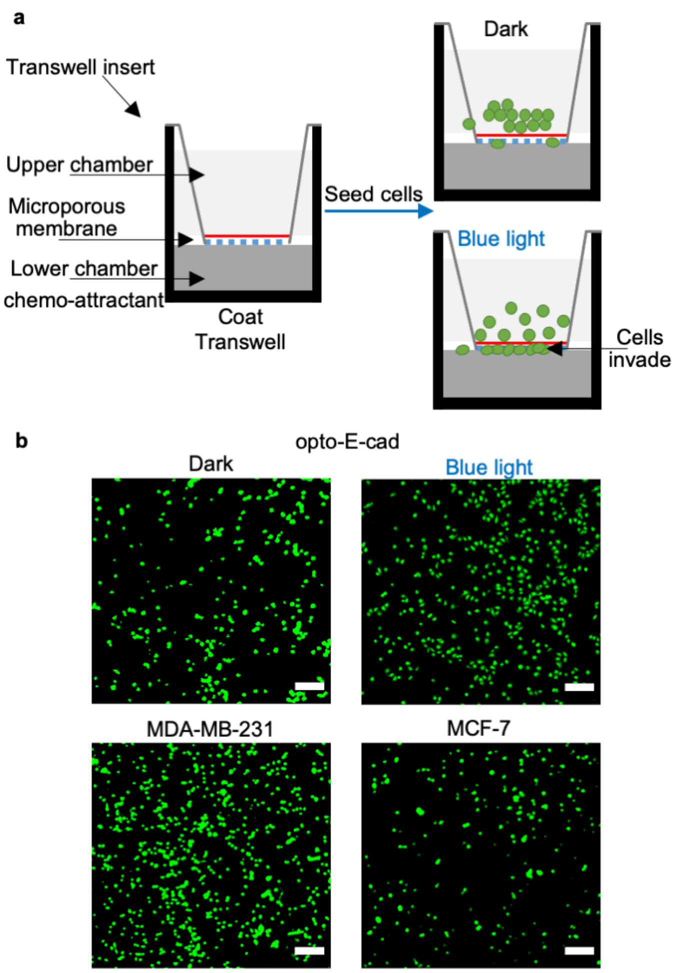

Supplementary Fig. 9 | Transwell migration assay. a, Workflow of Transwell migration assay. Cells are seeded in the upper chamber and migrate through the microporous membrane into the lower chamber. **b,** Fluorescence microscopy image of invaded cells in the lower Transwell chamber, where cell nuclei were stained with Hoechst dye (shown in green). Scale bars are 200 µm. Quantification in Fig. 5e.

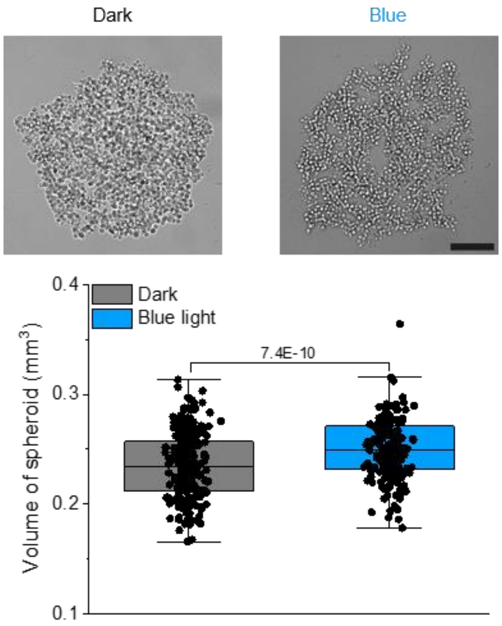

Supplementary Fig. 10 | Spheroid formation assay with opto-E-cad-A431D cells. Bright field images of spheroids formed from opto-E-cad-A431D cells in the dark and under blue light after 24 h. Scale bar is 200 µm. Spheroid volume after 24 h (two-tailed t-test, n= 224 (dark) and 178 (blue light) from 3 individual experiments).

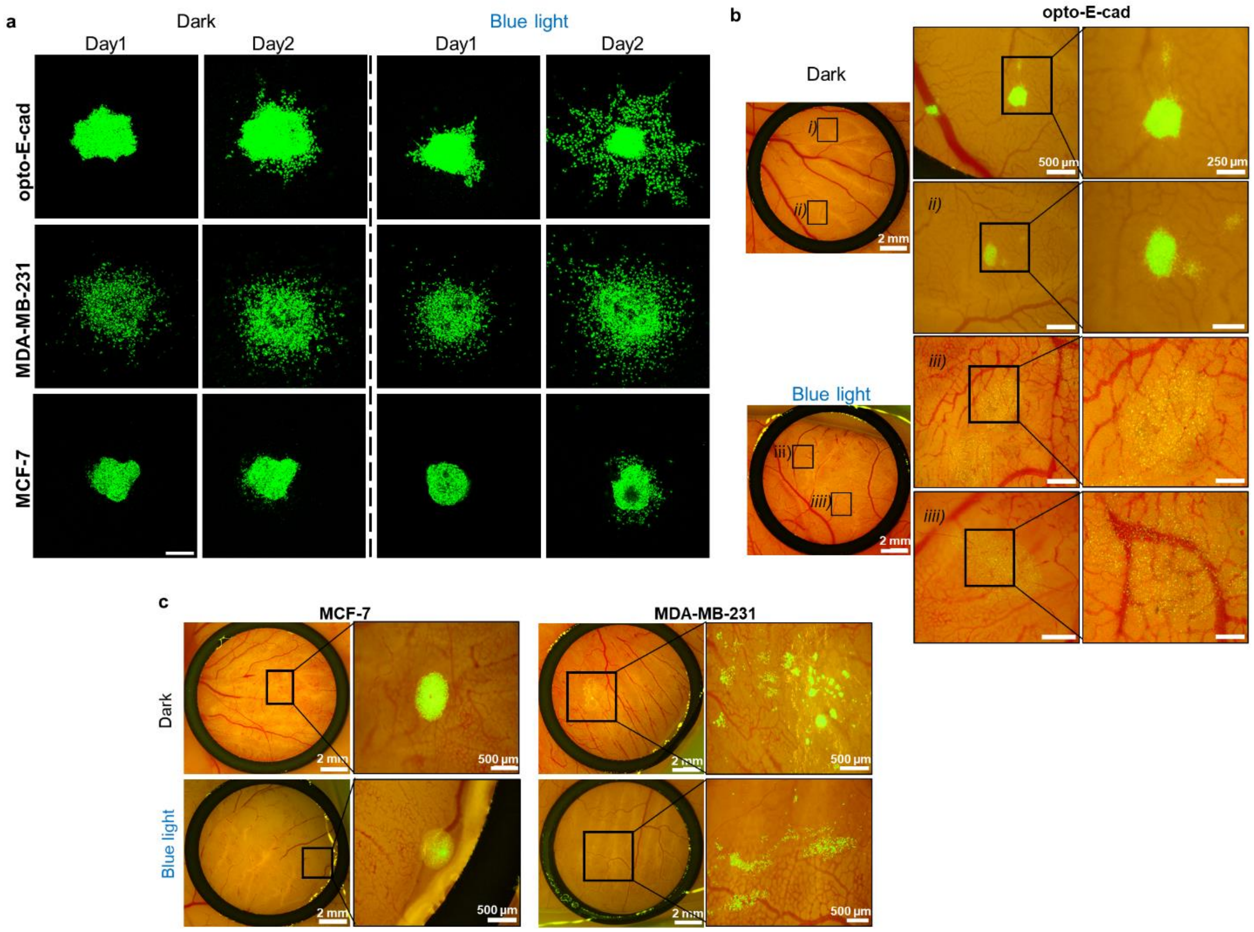

Supplementary Fig. 11 | Light controls cell invasion. a, Fluorescence microscopy images of spheroids from different cell types (shown in green) embedded in type I collagen gels in the dark and under blue light for 2 days (n=6). Scale bar is 200 µm. Fluorescence (shown in green) and bright field images of spheroids inoculated into CAM **b,** from opto-E-cad-MDA cells (n=3) and **c,** from wild-type MDA-MB-231 and MCF-7 cells in the dark and under blue light after 24 h (n=2).

MATLAB code: correlation length

```
function [C,u_comp]=CorLength(u_comp,v_comp)
clc
[L,W]=size(u_comp{1});
unit=356/(W+1); %horizontal is always 738.4 (1136px) um and the grid divides
it into W+1 segments
X=zeros(L,W);
Y=zeros(L,W);
C=[];
E=zeros(L,W);
u_meanSub=zeros(length(u_comp),1);
rho=zeros(L,W);

for i=25:length(u_comp)
[~,rho]=cart2pol(u_comp{i},v_comp{i});
for j=1:L
for k=1:W
if abs(rho(j,k))<3E-9
u_comp{i}(j,k)=NaN;
end
end
end
u_meanSub(i)=nanmean(nanmean(u_comp{i}));
end
u_mean=nanmean(u_meanSub);
for t=30:length(u_comp)-6
     u_compSub=((u_comp{t-6}+u_comp{t-5}+u_comp{t-4}+u_comp{t-3}+u_comp{t-
2}+u_comp{t-
1}+u_comp{t}+u_comp{t+1}+u_comp{t+2}+u_comp{t+3}+u_comp{t+4}+u_comp{t+5}+u_co
mp{t+6})/13)-u_mean;
for i=1:L
for j=1:W
for k=1:L
for m=1:W
Out=((u_compSub(i,j)).*(u_compSub(k,m)))/sqrt(((u_compSub(i,j))^2).*((u_compS
ub(k,m))^2));
if Out<0.1
R=abs(cart2pol(i-k,j-m))*unit;
%X(i,j)=k;
%Y(i,j)=m;
%k=L;
% m=W;
if R<E(i,j)&&E(i,j)~=0
E(i,j)=R;
elseif E(i,j)==0
E(i,j)=R;
end
end
end
end
% [~,E(i,j)]=cart2pol(X(i,j),Y(i,j));
%E(i,j)=E(i,j)*unit;
if E(i,j)==0
E(i,j)=NaN;
end
```

```
end
end
C=cat(1,C,E(:));
end
C=rmmissing(C);
end
```

MATLAB code: migration angle

```
function [thetaString,rhoString,thetaComp]=vectorPlot(u_filtered,v_filtered)
clc
rhoString=zeros(1,1);
thetaString=zeros(1,1);
thetaComp=zeros(1,1);
for j=1:length(u_filtered)
theta,rho]=cart2pol(u_filtered{j},v_filtered{j});
theta=reshape(theta.',1,[]);
rho=reshape(rho.',1,[]);
theta=theta-pi()/2;
rhoString=[rhoString -rho];
thetaString=[thetaString theta];
for i=1:length(theta)
if theta(i)<0
theta(i)=theta(i)+2*pi();
end
if theta(i)>pi()&&theta(i)<3/2*pi()
theta(i)=theta(i)-pi()/2;
elseif theta(i)>3/2*pi()
theta(i)=theta(i)+pi/2;
end
theta(i)=pi()-theta(i);
thetaComp=[thetaComp theta(i)];
end
end
thetaString(1)=[];
rhoString(1)=[];
thetaComp(1)=[];
figure
polarscatter(thetaString,rhoString,7,'b','filled') % 5 refers to size, and
'k' is color k=black b=blue
figure
polarhistogram(thetaComp,12,'FaceColor','blue');
f=gca;
f.ThetaLim=[0 180];
end
```

opto-E-cad protein sequence

White: Human E-cad
Yellow: Propeptide of E-cadherin cleaved after translation
Cyan: AsLOV2 domain
Green: GFP tag

```
MGPWSRSLSALLLLLQVSSWLCQEPEPCHPGFDAESYTFTVPRRHLERGRVLGRVNFEDCTGRQRTAYFSLDTRFKV
GTDGVITVKRPLRFHNPQIHFLVYAWDSTYRKFSTKVTLNTVGHHHRPPPHQASVSGIQAELLTFPNSSPGLRRQKR
DWVIPPISCPENEKGPFPKNLVQIKSNKDKEGKVFYSITGQGADTPPVGVFIIERETGWLKVTEPLDRERIATYTLF
SHAVSSNGNAVEDPMEILITVTDQNDNKPEFTQEVFKGSVMEGALPGTSVMEVTATLATTLERIEKNFVITDPRLPD
NPIIFASDSFLQLTEYSREEILGRNCRFLQGPETDRATVRKIRDAIDNQTEVTVQLINYTKSGKKFWNLFHLQPMRD
QKGDVQYFIGVQLDGTEHVRDAAEREGVMLIKKTAENIDEADADDDVNTYNAAIAYTILSQDPELPDKNMFTINRNT
GVISVVTTGLDRESFPTYTLVVQAADLQGEGLSTTATAVITVTDTNDNPPIFNPTTYKGQVPENEANVVITTLKVTD
ADAPNTPAWEAVYTILNDDGGQFVVTTNPVNNDGILKTAKGLDFEAKQQYILHVAVTNVVPFEVSLTTSTATVTVDV
LDVNEAPIFVPPEKRVEVSEDFGVGQEITSYTAQEPDTFMEQKITYRIWRDTANWLEINPDTGAISTRAELDREDFE
HVKNSTYTALIIATDNGSPVATGTGTLLLILSDVNDNAPIPEPRTIFFCERNPKPQVINIIDADLPPNTSPFTAELT
HGASANWTIQYNDPTQESIILKPKMALEVGDYKINLKLMDNQNKDQVTTLEVSVCDCEGAAGVCRKAQPVEAGLQIP
AILGILGGILALLILILLLLLFLRRRAVVKEPLLPPEDDTRDNVYYYDEEGGGEEDQDFDLSQLHRGLDARPEVTRN
DVAPTLMSVPRYLPRPANPDEIGNFIDENLKAADTDPTAPPYDSLLVFDYEGSGSEAASLSSLNSSESDKDQDYDYL
NEWGNRFKKLADMYGGGEDDPRSKGEELFTGVVPILVELDGDVNGHKFSVSGEGEGDATYGKLTLKFICTTGKLPVP
WPTLVTTLTYGVQCFSRYPDHMKQHDFFKSAMPEGYVQERTIFFKDDGNYKTRAEVKFEGDTLVNRIELKGIDFKED
GNILGHKLEYNYNSHNVYIMADKQKNGIKVNFKIRHNIEDGSVQLADHYQQNTPIGDGPVLLPDNHYLSTQSALSKD
PNEKRDHMVLLEFVTAAGITLGMDELIK-
```

opto-E-cad DNA sequence

```
ATGGGCCCTTGGAGCCGCAGCCTCTCGGCGCTGCTGCTGCTGCTGCAGGTCTCCTCTTGGCTCTGCCAGGAGCCGGA
GCCCTGCCACCCTGGCTTTGACGCCGAGAGCTACACGTTCACGGTGCCCCGGCGCCACCTGGAGAGAGGCCGCGTCC
TGGGCAGAGTGAATTTTGAAGATTGCACCGGTCGACAAAGGACAGCCTATTTTTCCCTCGACACCCGATTCAAAGTG
GGCACAGATGGTGTGATTACAGTCAAAAGGCCTCTACGGTTTCATAACCCACAGATCCATTTCTTGGTCTACGCCTG
GGACTCCACCTACAGAAAGTTTTCCACCAAAGTCACGCTGAATACAGTGGGGCACCACCACCGCCCCCCGCCCCATC
AGGCCTCCGTTTCTGGAATCCAAGCAGAATTGCTCACATTTCCCAACTCCTCTCCTGGCCTCAGAAGACAGAAGAGA
GACTGGGTTATTCCTCCCATCAGCTGCCCAGAAAATGAAAAAGGCCCATTTCCTAAAAACCTGGTTCAGATCAAATC
CAACAAAGACAAAGAAGGCAAGGTTTTCTACAGCATCACTGGCCAAGGAGCTGACACACCCCCTGTTGGTGTCTTTA
TTATTGAAAGAGAAACAGGATGGCTGAAGGTGACAGAGCCTCTGGATAGAGAACGCATTGCCACATACACTCTCTTC
TCTCACGCTGTGTCATCCAACGGGAATGCAGTTGAGGATCCAATGGAGATTTTGATCACGGTAACCGATCAGAATGA
CAACAAGCCCGAATTCACCCAGGAGGTCTTTAAGGGGTCTGTCATGGAAGGTGCTCTTCCAGGAACCTCTGTGATGG
AGGTCACAGCCACACTGGCGACCACCCTGGAACGTATTGAAAAGAACTTTGTGATTACCGATCCGCGTCTGCCGGAT
AACCCGATTATTTTTGCGAGCGATAGCTTTCTGCAGCTGACCGAATATAGCCGTGAAGAAATTCTGGGCCGTAACTG
CCGTTTTCTGCAGGGCCCGGAAACCGATCGTGCGACCGTGCGTAAAATTCGTGATGCGATTGATAACCAGACCGAAG
TGACCGTGCAGCTGATTAACTATACCAAAAGCGGCAAAAAATTTTGGAACCTGTTTCATCTGCAGCCGATGCGTGAT
CAGAAAGGCGATGTGCAGTATTTTATTGGCGTGCAGCTGGATGGTACCGAACATGTGCGTGATGCGGCGGAACGTGA
AGGCGTGATGCTGATCAAGAAAACCGCGGAAAACATTGATGAAGCGGACGCGGACGATGATGTGAACACCTACAATG
CCGCCATCGCTTACACCATCCTCAGCCAAGATCCTGAGCTCCCTGACAAAAATATGTTCACCATTAACAGGAACACA
GGAGTCATCAGTGTGGTCACCACTGGGCTGGACCGAGAGAGTTTCCCTACGTATACCCTGGTGGTTCAAGCTGCTGA
CCTTCAAGGTGAGGGGTTAAGCACAACAGCAACAGCTGTGATCACAGTCACTGACACCAACGATAATCCTCCGATCT
TCAATCCCACCACGTACAAGGGTCAGGTGCCTGAGAACGAGGCTAACGTCGTAATCACCACACTGAAAGTGACTGAT
GCTGATGCCCCCAATACCCCAGCGTGGGAGGCTGTATACACCATATTGAATGATGATGGTGGACAATTTGTCGTCAC
CACAAATCCAGTGAACAACGATGGCATTTTGAAAACAGCAAAGGGCTTGGATTTTGAGGCCAAGCAGCAGTACATTC
TACACGTAGCAGTGACGAATGTGGTACCTTTTGAGGTCTCTCTCACCACCTCCACAGCCACCGTCACCGTGGATGTG
CTGGATGTGAATGAAGCCCCCATCTTTGTGCCTCCTGAAAAGAGAGTGGAAGTGTCCGAGGACTTTGGCGTGGGCCA
```

GGAAATCACATCCTACACTGCCCAGGAGCCAGACACATTTATGGAACAGAAAATAACATATCGGATTTGGAGAGACA
CTGCCAACTGGCTGGAGATTAATCCGGACACTGGTGCCATTTCCACTCGGGCTGAGCTGGACAGGGAGGATTTTGAG
CACGTGAAGAACAGCACGTACACAGCCCTAATCATAGCTACAGACAATGGTTCTCCAGTTGCTACTGGAACAGGGAC
ACTTCTGCTGATCCTGTCTGATGTGAATGACAACGCCCCCATACCAGAACCTCGAACTATATTCTTCTGTGAGAGGA
ATCCAAAGCCTCAGGTCATAAACATCATTGATGCAGACCTTCCTCCCAATACATCTCCCTTCACAGCAGAACTAACA
CACGGGGCGAGTGCCAACTGGACCATTCAGTACAACGACCCAACCCAAGAATCTATCATTTTGAAGCCAAAGATGGC
CTTAGAGGTGGGTGACTACAAAATCAATCTCAAGCTCATGGATAACCAGAATAAAGACCAAGTGACCACCTTAGAGG
TCAGCGTGTGTGACTGTGAAGGGGCCGCCGGCGTCTGTAGGAAGGCACAGCCTGTCGAAGCAGGATTGCAAATTCCT
GCCATTCTGGGGATTCTTGGAGGAATTCTTGCTTTGCTAATTCTGATTCTGCTGCTCTTGCTGTTTCTTCGGAGGAG
AGCGGTGGTCAAAGAGCCCTTACTGCCCCCAGAGGATGACACCCGGGACAACGTTTATTACTATGATGAAGAAGGAG
GCGGAGAAGAGGACCAGGACTTTGACTTGAGCCAGCTGCACAGGGGCCTGGACGCTCGGCCTGAAGTGACTCGTAAC
GACGTTGCACCAACCCTCATGAGTGTCCCCCGGTATCTTCCCCGCCCTGCCAATCCCGATGAAATTGGAAATTTTAT
TGATGAAAATCTGAAAGCGGCTGATACTGACCCCACAGCCCCGCCTTATGATTCTCTGCTCGTGTTTGACTATGAAG
GAAGCGGTTCCGAAGCTGCTAGTCTGAGCTCCCTGAACTCCTCAGAGTCAGACAAAGACCAGGACTATGACTACTTG
AACGAATGGGGCAATCGCTTCAAGAAGCTGGCTGACATGTACGGAGGCGGCGAGGACGACCCGCGGAGCAAGGGGGA
GGAGCTGTTCACCGGGGTGGTGCCCATCCTGGTCGAGCTGGACGGCGACGTAAACGGCCACAAGTTCAGCGTGTCCG
GCGAGGGCGAGGGCGATGCCACCTACGGCAAGCTGACCCTGAAGTTCATCTGCACCACCGGCAAGCTGCCCGTGCCC
TGGCCCACCCTCGTGACCACCCTGACCTACGGCGTGCAGTGCTTCAGCCGCTACCCCGACCACATGAAGCAGCACGA
CTTCTTCAAGTCCGCCATGCCCGAAGGCTACGTCCAGGAGCGCACCATCTTCTTCAAGGACGACGGCAACTACAAGA
CCCGCGCCGAGGTGAAGTTCGAGGGCGACACCCTGGTGAACCGCATCGAGCTGAAGGGCATCGACTTCAAGGAGGAC
GGCAACATCCTGGGGCACAAGCTGGAGTACAACTACAACAGCCACAACGTCTATATCATGGCCGACAAGCAGAAGAA
CGGCATCAAGGTGAACTTCAAGATCCGCCACAACATCGAGGACGGCAGCGTGCAGCTCGCCGACCACTACCAGCAGA
ACACCCCCATCGGCGACGGCCCCGTGCTGCTGCCCGACAACCACTACCTGAGCACCCAGTCCGCCCTGAGCAAAGAC
CCCAACGAGAAGCGCGATCACATGGTCCTGCTGGAGTTCGTGACCGCCGCCGGGATCACTCTCGGCATGGACGAGCT
GATCAAG TAA

Primers:

Ecad T133 LOV2 FWD:

GTGATGGAGGTCACAGCCACACTGGCGACCACCCTGGAACG

Ecad T133 LOV2 REV:

CGTTCCAGGGTGGTCGCCAGTGTGGCTGTGACCTCCATCAC

LOV2 Ecad T133 FWD:

CGGAAAACATTGATGAAGCGGACGCGGACGATGATGTGAAC

LOV2 Ecad T133 REV:

GTTCACATCATCGTCCGCGTCCGCTTCATCAATGTTTTCCG
