# Supplementary material for: Reversible photoregulation of cell-cell adhesions with opto-E-cadherin
Source: Nat Commun. 2023 Oct 9;14:6292. doi: 10.1038/s41467-023-41932-0 (PMC10562482; doi:10.1038/s41467-023-41932-0)
Supplement: Supplementary file 4 — Description of additional supplementary files [file 41467_2023_41932_MOESM4_ESM.pdf]

Description Of Additional Supplementary Files

Supplementary Movie 1: Wound healing assay of opto-E-cad-MDA in the dark.

Supplementary Movie 2: Wound healing assay of opto-E-cad-MDA under blue light.
